# Cost-efficient and Custom Electrode-holder Assembly Infrastructure for EEG Recordings

**DOI:** 10.3390/s19194273

**Published:** 2019-10-02

**Authors:** Yuan-Pin Lin, Ting-Yu Chen, Wei-Jen Chen

**Affiliations:** 1 Laboratory for Neuroergonomics, Institute of Medical Science and Technology, National Sun Yat-sen University, Kaohsiung 80424, Taiwan; tychen@imst.nsysu.edu.tw (T.-Y.C.); wjc@imst.nsysu.edu.tw (W.-J.C.); 2 Department of Computer Science and Engineering, National Sun Yat-sen University, Kaohsiung 80424, Taiwan

**Keywords:** mobile EEG recordings, montage-replaceable headsets, BCI

## Abstract

Mobile electroencephalogram (EEG)-sensing technologies have rapidly progressed and made the access of neuroelectrical brain activity outside the laboratory in everyday life more realistic. However, most existing EEG headsets exhibit a fixed design, whereby its immobile montage in terms of electrode density and coverage inevitably poses a great challenge with applicability and generalizability to the fundamental study and application of the brain-computer interface (BCI). In this study, a cost-efficient, custom EEG-electrode holder infrastructure was designed through the assembly of primary components, including the sensor-positioning ring, inter-ring bridge, and bridge shield. It allows a user to (re)assemble a compact holder grid to accommodate a desired number of electrodes only to the regions of interest of the brain and iteratively adapt it to a given head size for optimal electrode-scalp contact and signal quality. This study empirically demonstrated its easy-to-fabricate nature by a low-end fused deposition modeling (FDM) 3D printer and proved its practicability of capturing event-related potential (ERP) and steady-state visual-evoked potential (SSVEP) signatures over 15 subjects. This paper highlights the possibilities for a cost-efficient electrode-holder assembly infrastructure with replaceable montage, flexibly retrofitted in an unlimited fashion, for an individual for distinctive fundamental EEG studies and BCI applications.

## 1. Introduction

Mobile electroencephalogram (EEG)-sensing technologies has rapidly progressed and made the access of neuroelectrical brain activity outside the laboratory in everyday life more realistic. The integrity of mobile systems by various manufacturers has been thoroughly validated and reported [1,2,3]. Owing to its interesting advantages of price, usability, and mobility, a considerable number of recent studies has emerged highlighting the aforementioned advantages to encourage the study of EEG correlates of brain states and functions in multidisciplinary domains, such as preliminary perception (e.g., visual/auditory responses [4,5,6]), cognition capacity (e.g., attention relocation [7,8], face recognition [1], and memory processing [9,10]), and psychological reaction (e.g., affective response [11,12], mental fatigue [13], and working stress [14]). The exploited state-/task-related EEG associations can be further leveraged with machine learning to underpin a mobile brain-computer interface (BCI) for a vast range of applications, such as rehabilitative exoskeleton control [15], visual function loss [16], attentional responses [17], lapse mitigation [18], quadcopter control [19], and visual-search games [20]. To this end, a mobile EEG device that enables a valid and reliable measurement of EEG signals over the brain is highly demanded and can play a vital role toward mobile BCI applications in real-world scenarios. 

Several research-grade and consumer-oriented mobile EEG devices are available in the market, such as Quick/Mobile series (Cognionics, Inc., San Diego, CA, USA), ENOBIO series (Neuroelectrics, Barcelona, Spain), Smarting (mBrainTrain, Inc., Belgrade, Serbia), Ultracortex (OpenBCI, Inc., Brooklyn, New York, USA), EPOC (Emotiv, Inc., San Francisco, California, USA), and MindWare (NeuroSky, Inc., San Jose, CA, USA). Their general technical specifications or other devices can be referred to [2,8,21]. As compared to conventional bulk, tethered systems in laboratory, the mobile system shares common features with miniaturized light-weight amplifier, wireless telemetry, and/or dry/gel/saline electrodes, facilitating the applicability of EEG recording with more naturalistic settings (e.g., non-stationary subjects [5,17]). Aside from commercial products, continuous research effort has been invested in hardware innovation from the aspects of enhancing signal quality, device usability and mobility, cost efficiency, and practical applicability. Most endeavors have been directed to the counterparts of dry electrode [6,22,23,24,25,26,27,28], signal amplifier [4,8,22,29,30,31], and standalone computing [30]. Many commercial or customized mobile devices can be useful and provide acceptable/promising signal quality for specific circumstances. Along with signal validity and reliability, the selection of a mobile device should also consider the electrode montage in terms of the number of electrodes and their positions on the scalp [21]. It is essential to confirm whether its headset coverage offers signal accessibility to the brain regions of interest in an EEG study as well as a BCI task that is to be performed. Given the diversity of manufacturers, electrode density and coverage vary widely, from one single electrode over a single brain region (e.g., NeuroSky MindWare) to high-density electrodes with a whole-brain coverage (e.g., Cognionics Mobile-64/-128). A low-density example may constrain usability because of the sparseness or absence of electrodes over certain brain regions (e.g., Emotiv EPOC does not contain midline electrodes), whereas a high-density example can provide an ideal setting but inevitably poses a financial barrier and time-consuming preparation. The most critical issue is that a whole-brain coverage is plausibly with the presence of a large number of redundant electrodes as long as a specific BCI task is concerned, such as non-posterior electrodes for steady-state visual-evoke potential (SSVEP) [32] and non-sensorimotor electrodes for motor imagery (MI) [33]. The electrode placements for both the low- and high-density headsets are typically designed in an immobile manner. As such, customized headsets made of auxiliary cables, rack/stripe, headband, or soft/elastic cap have been used to secure electrodes for a specific application [8,18,20,26,33,34]. Up until recently, OpenBCI Ultracortex Mark series has gained much attention in the community and can be considered as the state-of-the-art headset frame. It enables to change the montage to a certain extent by voluntarily placing a desired number of electrodes on to a fixed, 3D-printed 35-location frame. Yet, the headset may have redundant electrode holders in use. Moreover, the design of a rigid frame or soft cap in commercial products does not ideally fit well to distinctive head sizes and shapes of individuals in different age groups. A feasible remedy is to offer a headset with different sizes, suitable for a desired range of head circumference (e.g., OpenBCI Ultracortex Mark series and Neuroelectrics ENOBIO series). It is reasonably expected that an inappropriate headset size may compromise the sensor locations and their contact to the scalp, thereby downgrading signal quality to a certain extent. Taken together, a headset with an immobile sensor-holder structure of electrode density and coverage may pose a great challenge with applicability and generalizability to both commercial and custom mobile EEG devices. To date, headsets in the market do not offer a flexible headset frame with a customable montage design. 

Therefore, in this study, an electrode-holder assembly infrastructure for EEG recordings was developed, which allows a user to (re)assemble a compact holder grid to accommodate a desired number of electrodes to the brain regions of interest in an unlimited fashion, in accordance to the EEG and BCI study of interest to be performed. During the assembly, the formed headset can iteratively adapt to a given head size and shape, practically ensuring electrode-scalp contact and signal quality. All the conceived assembly elements (e.g., sensor-positioning ring, inter-ring bridge, and bridge shield) were manufactured via a low-end consumer 3D printer and the practicability of the assembled headset was validated through the study of event-related potentials (ERP) and steady-state visual evoked potential (SSVEP) tasks. This study demonstrated exceptional possibilities for a cost-efficient electrode-holder assembly infrastructure with replaceable montage retrofitted for an individual in unlimited manner, for distinctive fundamental EEG investigations and BCI applications.

## 2. Materials and Methods

### 2.1. Design and Practice of Sensor-holder Assembly Infrastructure

The assembly infrastructure developed in this study has the following practical benefits for EEG recording as well as BCI deployment. First, the infrastructure allows (re)assembling without limit to accommodate a desired number of electrodes over brain regions of interest, leading to a compact headset without the need for redundant electrodes attached to the head (i.e., saving cost, weight, and calibration time, and improving wearability and comfort). In addition, the infrastructure is capable of optimally retrofitting the headset to a certain head size/circumference for each individual during assembly. This provides the advantage of not only positioning the sensors over the target brain regions as consistently as possible for different age groups in the same study or task but also ensuring the attached electrodes to be in good contact with the scalp for better signal quality. As such, the proposed infrastructure can be considered a design of multi-purpose, with a montage-replaceable headset to record EEG signals from only the brain regions of interest.

**Figure 1:**
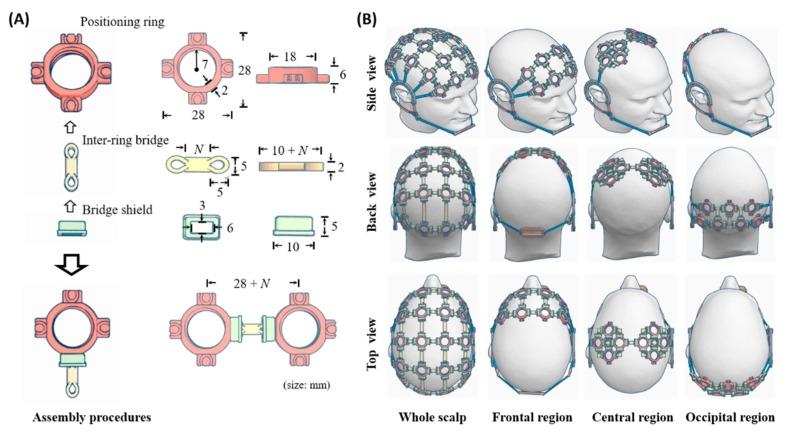
Illustration of the developed electrode-holder assembly infrastructure. (**A**) Three primary components and their assembly procedures. The sizes are in millimeter (*N* = 11, 13, 40, and 50 used in this work); (**B**) four assembly embodiments with coverages of the entire scalp and individual regions of interest (simulated by 3D design software).

The assembled infrastructure was conceived to compose of three primary components including sensor positioning ring, inter-ring bridge, and bridge shield (as shown in Figure 1A). The positioning ring is used to place an EEG electrode, whereas the inter-ring bridge is utilized to assemble a plurality of the rings in a desired montage. The bridge shield covers an assembled junction of the ring and bridge, thereby fastening the connection (i.e., prevent them from falling apart in use). The positioning ring can be fabricated to the required shape and size to comply with the specifications of different EEG sensors. The present form was designed to better accommodate the adopted dry flex electrodes (Cognionics, Inc., San Diego, CA, USA). Analogously, the inter-ring bridge can work in different lengths and curvatures, such that the assembly holders naturally form a desired 3D grid to fit the head size. The bridge and shield were designed empirically to be able to quickly assemble positioning rings to be connected or disassemble them. By leveraging supplementary counterparts of ear and chin strips and holders, a user can easily wear an assembled headset on his/her head or disassemble it. Figure 1B illustrates four plausible embodiments of the proposed holder assembly (simulated by 3D design software) to measure the EEG signals from the entire scalp or individual brain regions of interest, e.g., frontal, sensorimotor, and occipital parts. 

**Figure 2:**
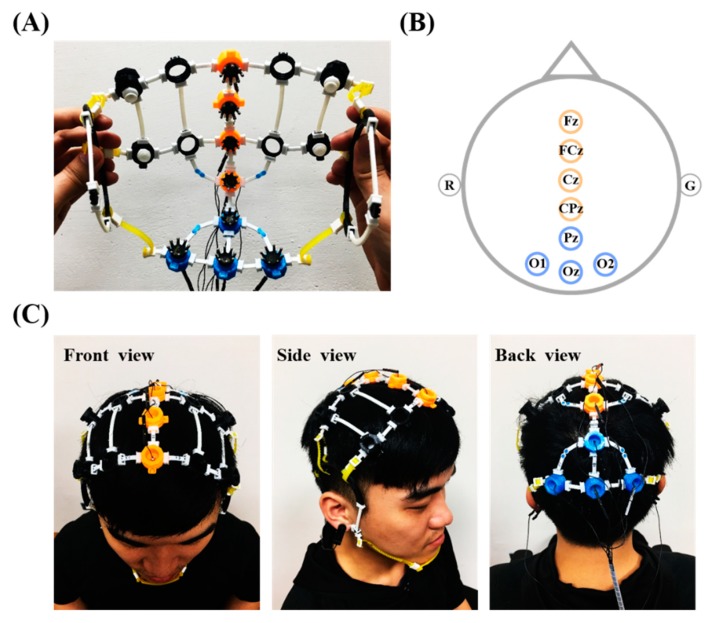
Implementation of an 8-electrode holder assembly grid. (**A**) Assembled headset embedded with Cognionics dry electrodes; (**B**) 8-channel montage; (**C**) headset wore on the head at different view angles.

To demonstrate the practicality, an 8-electrode holder grid was implemented to accommodate EEG electrodes over the fronto-central midline and parieto-occipital regions (four for each region, as shown in Figure 2A), in accordance with the signal amplifier and experimental tasks employed in this study (described in the later subsections). Note that a supplementary component of tiny bridge extender (blue) was also implemented for the assembly demonstration. The extender shows the capability to prolong a bridge by concatenating multiples bridges if the bridge in a desired length is unavailable. All the assembled components were fabricated by a low-end fused deposition modeling (FDM) 3D printer (layer resolution of 0.3–0.4 mm applied with 100% infill) using polylactide (PLA) or ethylene vinyl acetate (EVA) filaments. The PLA was used to print the stiff positioning ring, whereas the EVA was used to print the bridge and shield in a bendable/flexible manner. The printed components could work supposedly as long as possible in normal use. The Cognionics dry flex electrodes, which were made by Ag/AgCl-covered elastomer, were adopted for EEG measurement. Referring to Figure 2B, the midline locations include Fz, FCz, Cz, and CPz (orange rings), whereas the posterior counterparts include Pz, O1, Oz, and O2 (blue rings). A few auxiliary rings (black), embedding non-conductive comfort pads, were also assembled to further improve wearability. Figure 2C demonstrates the practice of the assembled 8-ch EEG headset wore on an adult head at different view angles. In the present demonstration, the positioning ring inserted a supplementary component of electrode adapter to snap the dry electrode via snap fastener. That is, the adaptor can be made in various forms to accommodate desired electrodes in the market. In detail, the 8-electrode grid was assembled within around 15 minutes by 16 rings (electrode: 8, comfort pad: 8), 27 bridges (11 mm: 14, 13 mm: 9, 40 mm: 2, 50 mm: 2), and 54 shields, which cost less than 5 USD for the filaments. 

### 2.2. EEG Recording

In this study, a portable, custom 8-ch EEG-sensing device [8] was employed to wire the assembled headset to measure the EEG signals. This device had been systematically validated by conducting a simultaneous EEG recording of three subjects in 10 day sessions. Successful measurement of weak, time-locked, and phase-locked ERP signals demonstrated its proficiency in terms of signal quality, reliability, and data-event synchronization. Briefly, its major technical specifications are as follows. This device was designed to measure up to eight channels of analog signal input, filter the signals in a bandwidth of 0.6–56.5 Hz, sample and quantize the signals at 250 Hz with a resolution of 10 bits, and wirelessly transmit the digitized streams (synchronized with events if any) via a typical Bluetooth communication protocol. More details can be found in [8] regarding hardware implementation and verification. The left and right earlobes were set to reference and ground sites, respectively, during recording. 

### 2.3. Participants and Experimental Paradigms

Fifteen healthy participants (six males and nine females; age 22.4 ± 1.4 years.) were recruited to validate the practicability of the assembled electrode-holder grid. They were all students from the Colleges of Science and of Engineering and had normal hearing and normal or corrected-to-normal vision. Participants gave their written informed consent approved by Human Research Protections Program of the local ethics committee and received monetary compensation.

In this study, two stereotypical paradigms were conducted underpinning different modalities of visual perception and cognitive processing through SSVEP and oddball ERP protocols, respectively. These two tasks allowed us to assess the signal quality in terms of time- and phase-locked temporal (i.e., ERP amplitude) and spectral (i.e., SSVEP amplitude) context while wearing the assembled headset. The four central midline electrodes were intended to capture not only the N100 ERP (i.e., negative deflection in the voltage around 100–170 ms after stimulation onset) of early sensory perception but also the P300 ERP (i.e., positive deflection peak by 300–500 ms) for the endogenous process of attentional relocation, engaging in an oddball paradigm [8,35,36]. On the other hand, the four parieto-occipital electrodes attempted to capture frequency-coded responses of SSVEPs originating from the visual cortex that are synchronized to continuous, repetitive visual stimulation [32]. The rationale for performing both tasks was that if the assembled electrode-holder grid cannot integrate its numerous components of the positioning rings, inter-ring bridge, and bridge shields in a proficient structure and position the desired electrodes in a non-uniform curved surface across the head (e.g., fronto-central midline and posterior parts), poor contact for the attached electrodes will result, barely returning the prominent ERP and SSVEP characteristics from the group of 15 participants. The detailed parameters and procedures for the ERP and SSVEP task are depicted as follows.

#### 2.3.1. Auditory Oddball ERP Paradigm

The oddball paradigm consisted of two pure tones in different occurrences. A high-pitched tone (1000 Hz) was infrequently formed (30%, called target tone) as compared to the frequently presented low-pitched tone (500 Hz, called standard tone). Both these tones lasted for 500 ms and were played through the speakers. Each participant was instructed to focus their gaze at the fixation cross located at the center on a 27” LCD monitor, attentively responding to the target tone by pressing a handheld button as fast as possible, while ignoring the frequent standard tone. The paradigm was composed of four blocks; each contained 100 trials with both tones in random order and with an inter-trial jitter of 0.5–1.5 s. The oddball paradigm for each participant corresponded to a total of 120 target trials versus 280 standard trials for sequential ERP analysis.

#### 2.3.2. SSVEP Paradigm

Three repetitive black/white visual flickers (frequency: 10, 12, and 14 Hz; size 5.5 cm × 5.5 cm) located at the vertices of a triangle were presented on a 27-inch LCD monitor. A fixation cross at the triangle center (no flickering) was also presented as control. The participant was instructed to attentively gaze at the fixation cross or at one of the three flickers for four seconds in turn. The paradigm contained four 20-trial blocks; each block presented one of four visual targets five times in a random order. An auditory cure guided the participant to shift their gaze to the next target within an inter-trial duration of 2.5 s. The SSVEP task collected 80 trials (20 per target) from each participant.

During the recording, each participant sat comfortably on a chair in a dimly lit room. They underwent both the aforementioned tasks on different days in a random order. Each task lasted approximately 30 min, which comprised experiment briefing, headset capping, and data recording. The assembled headset was cleaned simply by a wet wipe with a bit of sanitizer before the capping. During the capping, the initial inter-ring bridges with respect to certain positioning rings could be replaced by different lengths for certain individuals because of a distinctive head size. In the meanwhile, the participant was informed about mild to moderate pressure needed for attaching dry electrodes well to the scalp. This procedure typically took 5–10 min to confirm the comfort and ensure the contact between the fronto-central midline and/or parieto-occipital electrodes and the scalp. 

### 2.4. EEG Analysis

This study employed the following procedures to pre-process and analyze the collected ERP and SSVEP sessions, including (1) bandpass filtering (ERP: 1–30 Hz, SSVEP: 7–17 Hz), (2) trial segmentation (ERP: −0.2 to 1 s, SSVEP: 0 to 4 s), (3) trial analysis (ERP: synchronized averaging calculation, SSVEP: power spectral density (PSD) estimation, and (4) signal-to-noise (SNR) calculation on the peaks of interest (ERP: P300 amplitude, SSVEP: 10-, 12-, and 14-Hz amplitude). Other signal modality-specific parameters or steps are depicted below. Data preprocessing, analysis, and visualization were performed using the open source EEGLab toolbox/scripts [37] and self-custom scripts in MATLAB (The Mathworks, Inc., MA, USA). 

#### 2.4.1. ERP Analysis

Following trial segmentation, the trials containing extreme amplitudes (>±100 µV) or corresponding to erroneous behavioral responses were removed from further analysis (6.5 ± 0.1% trials discarded on average). In addition, the z-score standardization was applied to the averaged ERP profile of each individual prior to calculate the grand average ERP. The P300 SNR was derived by dividing the P300 peak amplitude (300–500 ms) by the standard deviation of the prestimulus baseline (−200 to 0 ms) as the procedure in [8,38]. Finally, a paired t-test was applied to assess the statistical difference in P300 SNR between the target versus non-target events. 

#### 2.4.2. SSVEP Analysis

Synchronized averaging calculation was also applied to the 4-s SSVEP trials corresponding to each of the visual targets after trial segmentation. The short-time Fourier transform (STFT) with 1-s Hamming window (with 80% overlap) was subsequently employed to estimate the EEG spectrogram with a frequency resolution of 1 Hz and yield the averaged PSDs across all time windows. Lastly, SSVEP SNR was defined by the ratio of spectral amplitude in the target frequency (10, 12, or 14 Hz) versus the mean amplitude of its six neighboring frequencies (i.e., three frequencies to each side), as referred to [39].

## 3. Results

**Figure 3:**
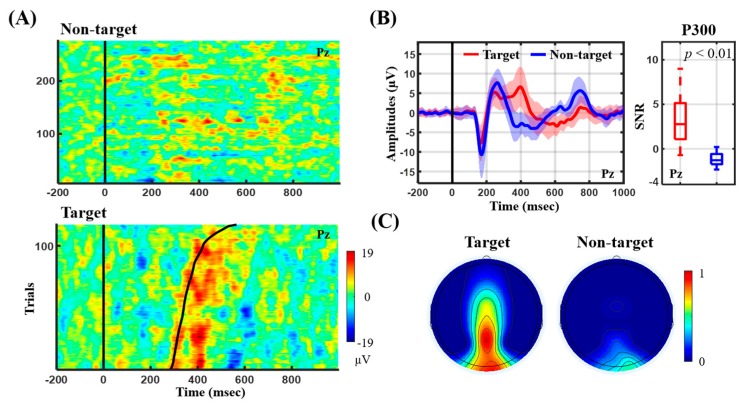
The ERP outcomes recorded in an auditory oddball ERP paradigm. (**A**) ERP images of Pz from a representative subject; (**B**) ERP profiles and P300 SNR (i.e., 300–500 ms) of Pz summarized by 15 participants; (**C**) averaged topographic mapping of P300 SNR using the adopted eight electrodes.

Figure 3 presents the ERP outcome in terms of its temporal profile and P300 SNR for the auditory oddball paradigm. Figure 3A shows the ERP image from the representative subject, whereas Figure 3B,C summarize the ERP profile and P300 SNR of the 15 participants. Note that the ERP image visualizes single ERP trials and sorts them by button-press response time (RT) in ascending order. From the representative target ERP image (see Figure 3A), the salient P300 peak closely followed the RT (black trace) with a distinct amplitude. A longer RT tended to accompany a weaker amplitude and prolonged latency. On the contrary, the non-target ERP image did not exhibit the P300 peak. For the group analysis (see Figure 3B), the target event (red profile) exclusively and reliably elicited the P300 peak (at ~400 ms) against the non-target event (blue profile) for 15 participants, leading to a statistical significance in P300 SNR (*p* < 0.01). The N100 peak (at ~170 ms) time-locked to the stimulus onset was also seen for both events. The topographic mapping of P300 SNR further showed that the highest value was located at Pz as compared to other electrodes for the target event only (see Figure 3C). 

**Figure 4:**
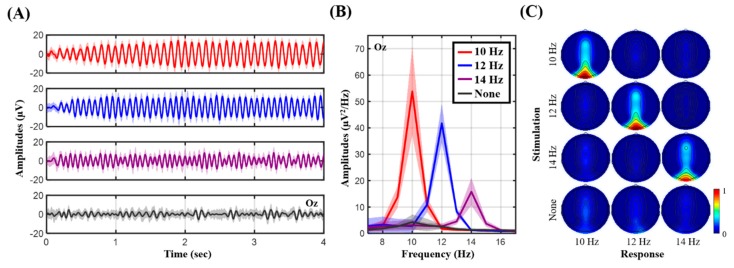
The SSVEP outcomes corresponding to frequency-coded visual flickers against fixation cross. (**A**) Temporal profiles of Oz from a representative subject; (**B**) spectral profiles of Oz from the same representative subject; (**C**) averaged 8-ch topographic mapping of SSVEP SNR using 4-s trials by 15 participants.

Figure 4 shows the measured SSVEP signatures as exposed to frequency-coded visual flickers. Figure 4A,B present the temporal and spectral profiles from the representative subject, where Figure 4C presents the topographic mapping of SNR values over the 15 participants. The single-subject temporal profile was clearly associated with neural activity synchronized to the onset of the visual flickers at 10, 12, and 14 Hz (see Figure 4A). Their spectral profiles also exhibited salient peaks dominated at the corresponding frequencies (see Figure 4B). Furthermore, the topographic SNR mapping in Figure 4C showed that the occipital electrodes accompanied higher SNR values reactive to each of the visual flickers as compared to other electrodes, especially at Oz. Unlike the frequency-modulated flickers, the eye-gaze at the fixation cross barely led to frequency-synchronized neural modulation. 

## 4. Discussion

In this study, an electrode-holder infrastructure was developed for EEG recordings through the assembly of three primary components, including sensor positioning ring, inter-ring bridge, and bridge shield. Given one assembly set, a user is able to self-assemble a headset with a desired montage to position electrodes at the brain regions of interest in unlimited manner for fundamental research as well as BCI applications. Whereas most existing headsets exhibit an immobile design, the proposed headset assembly accommodates the use of a compact set of electrodes placed over certain regions but removes other redundant electrodes or counterparts that are used for different purposes. During assembly, the formed 3D structure grid can be retrofitted iteratively to a given head size or shape of individuals in different age groups. In this way, the electrode-scalp contact can be optimized to ensure the signal quality accordingly as compared to that of an immobile headset with an unsuitable size. Most practically, this study demonstrated that the fabrication of the assembly structures can be accomplished using a low-cost FDM 3D printer, which is cheaper to custom manufacture than injection molding. Although the state-of-the-art OpenBCI Ultracortex Mark series is also a 3D-printed design and allows voluntarily placing the electrodes over default 35 locations, its headset frame is fixed (i.e., having redundant electrode holders in use) and only available in three head circumference sizes. Our assembly holder departed from the Ultracortex frame substantially with respect to unlimitedly (re)assembling of a compact holder grid for different purposes or even head sizes given one assembly set of the primary components (i.e., a LEGO-like headset) without additional cost. In addition, as assembling an Ultracortex-like headset frame, our design will cost only about 10 USD (using 35 rings, 58 bridges, and 116 shields) by PLA and EVA filaments. Aside from continuous innovation for electrode and amplifier technologies [4,6,8,22,23,24,25,26,27,28,29,30,31], this study contributed to a cost-efficient, montage-customable headset assembly infrastructure for a range of applications. It is worth noting that the proposed assembly grid has the capability of inserting a variety of commercial electrodes (i.e., electrode adaptor required) and wiring them to a commercial amplifier if a direct connection is feasible (e.g., OpenBCI Ganglion/Cyton biosensing board). 

To validate the practicability of the conceived electrode-holder assembly, this study implemented an 8-ch electrode-holder grid embedded with Cognionics dry electrodes for incorporation with a portable, custom EEG amplifier [8] for EEG recordings. Its electrode coverage over the fronto-central midline and parieto-occipital regions was intended to capture the prominent ERP P300 and frequency-modulated signal peaks while engaging in the auditory oddball and SSVEP tasks, respectively. Given the EEG recordings of the 15 participants, the resultant ERP and SSVEP outcomes were in line with those found in the literature. From the ERP perspective (c.f., Figure 3), the target tones predominantly aroused the attention-related P300 amplitude, with the highest SNR over the parietal region at Pz [8,35,36]. The single-subject example further demonstrated a clear RT-modulated P300 profile consistently in [8]. In addition, both auditory tones corresponded to the N100 component, early component of exogenous stimulus detection [8,36]. From the SSVEP perspective (c.f., Figure 4), the occipital electrodes exclusively recorded strong SSVEP amplitudes corresponding to the targeted frequencies (Oz with the highest SNR), as SSVEP responses originate from the visual cortex and are synchronized to frequency-coded visual stimulation [5,13]. Taken together, the replication of the time-locked and phase-locked ERP and SSVEP signatures empirically demonstrated the satisfactory electrode-scalp contact and signal quality while wearing the proposed headset assembly for EEG recordings. 

There are several possibilities or advantages far beyond the current demonstration of EEG recording. First, the sensor-holder ring can be fabricated with different specifications to organize either homogeneous/heterogeneous sensors or sensors and stimulators on the same headset, e.g., comparing EEG electrodes made by different materials at very adjacent sites [1,5,35,40], facilitating a simultaneous EEG and functional near-infrared spectroscopy (fNIR) recording over the brain regions of interest [31], and monitoring EEG activity while applying transcranial alternating current (TAC) stimulation [41]. In addition, the assembly structure is surely capable of being retrofitted to a head-mounted display system (e.g., AR/VR goggle), worn on the head for stand-alone application [16,19,42]. In contrast, a headset with an immobile design to an end user may make the above applications difficult to implement.

Although this study presented a promising start to a cost-efficient, montage-replaceable headset assembly in mobile EEG technologies, future effort should be devoted to further demonstrating and improving its robustness and generalizability. First, EEG recordings can be replicated on non-stationary subjects (e.g., walking on treadmill [5]). This will be a rigorous yet realistic setting to evaluate the behavior of the electrode-scalp contact under head movement conditions (e.g., bobbing or swaying) in walking. Furthermore, in constructing a truly multi-purpose assembly set with considerable wearability and comfort, it is essential to pay more attention to the mechanical properties of each of the assembled components, such as size, length, curvature, shape, material, and weight, and what should be included, at least for the capacity of retrofitting a desired montage from sparse to high-dense settings over distinct brain regions of interest. It is also helpful to have a guideline following the international 10–20 system of electrode placement. A holder grid can then be customized by selecting suitable components efficiently (e.g., inter-ring bridge noted with length) for distinctive brain regions of interest during assembly.

## 5. Conclusions

In this study, a cost-efficient, custom EEG-electrode holder infrastructure was developed through the assembly of primary components, including sensor positioning ring, inter-ring bridge, and bridge shield. For different studies or BCI tasks, the user can (re)assemble a compact holder grid in an unlimited fashion to accommodate a desired number of electrodes only to the brain regions of interest and iteratively adapt it to a given head size for optimal electrode-scalp contact and signal quality. This study empirically demonstrated the easy-to-fabricate nature of the infrastructure through a low-cost 3D printer and its practicability in measuring ERP and SSVEP signals was proven over studies with 15 participants. Through this study, a montage-replaceable headset tailored to be applicable for a variety of purposes was explored.
